# A decade-long longitudinal survey shows that the Supreme Court is now much more conservative than the public

**DOI:** 10.1073/pnas.2120284119

**Published:** 2022-06-06

**Authors:** Stephen Jessee, Neil Malhotra, Maya Sen

**Affiliations:** ^a^Department of Government, University of Texas, Austin, TX 78712;; ^b^Graduate School of Business, Stanford University, Stanford, CA 94305;; ^c^John F. Kennedy School of Government, Harvard University, Cambridge, MA 02138

**Keywords:** Supreme Court, ideology, representation

## Abstract

Has the US Supreme Court become more conservative than the public? We introduce results of three surveys conducted over the course of a decade that ask respondents about their opinions on the policy issues before the court. Using these data, we show that the gap between the court and the public has grown since 2020, with the court moving from being quite close to the average American to a position that is more conservative than the majority of Americans. Second, in contrast to findings showing consistency in the public’s approval of or deference to the court, we find that the public’s expectations of the court vary significantly over time and in tandem with changes in the court’s composition and recent rulings. Even so, many members of the public currently underestimate the court’s conservative leaning. Third, we find that respondents’ perceptions of the court’s ideology relative to their own are associated with support for institutional changes but with important differences between Democrats and Republicans. The fact that so many people currently underestimate how conservative the court is implies that support for proposed changes to the court may be weaker than it would be if people knew with greater accuracy the court’s conservative nature.

## Significance

Leveraging three unique surveys collected over a decade that ask members of the public about the policy issues before the US Supreme Court, we show how the court stands relative to the public. As we demonstrate, the court has, since 2020, become much more conservative than the public and is now more similar to Republicans in its ideological position on key issues. We also find that members of the public update their beliefs about the court’s ideology when its composition and rulings change. Even so, many members of the public currently underestimate the court’s conservative leaning, which in turn makes them less likely to support making changes to the institution than they would otherwise.

The US Supreme Court has undergone significant changes over the past decade and its rulings—including those involving key policy issues such as civil rights, health care, and abortion—have shifted along with its composition. Does this mean that the court is now out of step with public opinion? If so, by how much?

Such questions are important. The court lacks the power of “either the sword or the purse” (1) and so must draw its legitimacy as a governing institution from public support. For the most part, observers think that the court has rarely moved too far from public sentiment through either self-correction or from the back-and-forth of the appointments process (2, 3). However, in recent years, experts have questioned whether the court remains aligned with the public. Many believe that this puts the court at risk for being seen as behaving politically, which could have significant consequences. “If the public comes to see judges as merely ‘politicians in robes,”’ as Justice Stephen Breyer has written, “its confidence in the courts, and in the rule of law itself, can only decline” (4).

We provide a long-view examination of these questions by leveraging a unique set of three surveys taken over 12 y (in 2010, 2020, and 2021) that ask respondents their opinions on the actual policy questions before the court. By comparing respondents’ own preferences on these issues, their expectations about how the court will rule, and the court’s eventual rulings, we can estimate divergence between the court’s and the public’s ideological positioning and also address whether the public’s expectations match up with the court’s actual behavior. By ideological position, we mean that political views can be represented spatially as locations in one-dimensional Euclidean space (5).

Importantly, because our data were collected in the span of over a decade, we can also examine how these patterns have fluctuated over time, leveraging significant changes to the court’s composition—including two changes to the identity of the court’s median, and therefore pivotal, member (6). Further, collecting data over the short time period of 2020 and 2021 allows us to conduct a pre-post analysis of public opinion toward the court and how it may correspond with a sudden change in its ideological composition.

We make several discoveries that contribute to our understanding of the court’s ideological positioning and public perceptions. First, we document the empirical fact that the court’s rulings were once similar to the preferences of the average American but are now more conservative than the preferences of the majority of Americans. Second, we show that, unlike beliefs about the legitimacy of the court and its role in American democracy (which other research has shown remain consistently high), expectations about how the court will rule fluctuate remarkably. These fluctuations suggest that the public may be sensitive to the changing composition of the court as well as to its rulings: although much has changed since 2010, as the court has moved sharply to the right, so, too, have recent expectations of how the court will rule (7, 8). However, even so, many Americans underestimate the court’s conservative lean, with Democrats particularly likely to peg the court as more liberal than it actually is (9). Third, consistent with previous work (10), we find that expected ideological distance from the court is associated with support for changing institutional features of the court but that these relationships differ between Democrats and Republicans, particularly on the issue of court expansion. This suggests that if people—and particularly Democrats—knew with accuracy the court’s conservative nature, support for court curbing might increase.

The findings here also contribute to how scholars characterize changes in the court’s ideology. The most widely used approaches to estimating justice ideology rely solely on justices’ votes (11). However, such approaches face a fundamental difficulty in anchoring estimates of the court’s ideology over time. As the designers of these methods themselves point out, they take the case docket as given, when in fact the justices have substantial discretion over the cases they hear. It is not possible to detect if justices as a whole are moving ideologically or if the docket has changed—that is, if the justices simply hear more conservative or more liberal cases over time (12).

To be clear, our analyses also take the docket as given; however, our approach focuses on the relative comparison between the public and the court, particularly for the most publicly salient cases heard by the court each term. If the court hears different types of salient cases in a given term (e.g., cases on abortion and affirmative action instead of on gay rights and gun control), then its estimated position relative to the public will be driven more by these issues. As discussed in *SI Appendix*, however, there is evidence that the ideological dimension underlying the salient cases that make it to the court is structured in a relatively consistent way across survey waves. Further, the salient cases that we asked about seem to be similar to the entire docket in terms of estimating the ideology of both the justices and the public. Our method allows us to analyze how the court has shifted over time vis à vis public opinion. This enables us to ask a different but equally important substantive question: How has the Supreme Court’s ideology shifted relative to an important baseline—the views of the public at a given time?

It is also generally not possible to compare commonly used voting-based measures of judicial ideology to survey-based measures of public opinion as they are based on different quantities. The situation is analogous to trying to compare the abilities of groups of students who have taken two different examinations. Comparing the students’ grades to make inferences about relative abilities between these two groups is basically impossible unless one can reasonably assume that the two examinations are equally difficult.

Under our approach, ordinary citizens are “taking the same exam” as the justices by stating their views on the same subset of substantive issues. Although we use a nonrandom subset of cases (emphasizing issues that are likely to be both salient and understandable to the general public), the surveyed cases appear to be broadly representative of the entire docket as we discuss in *Results* and in *SI Appendix*. Further, we do not focus on jurisprudence or legal procedure but, instead, on substantive policy and politics. This allows us to see whether the concrete policy actions taken by the court (e.g., is abortion or affirmative action constitutional?) are in step with public opinion and, if not, how far and in which direction they have shifted.

After changes in the court’s composition over the last 10 y, for example, we find that the court is now sharply to the right of public opinion. We estimate that after the median shifted from John Roberts to Brett Kavanaugh in late 2020, the court is now near the typical Republican and to the ideological right of roughly three quarters of all Americans.

## Results

Each of the three surveys (fielded in 2010, 2020, and 2021) asked respondents their preferences on the major issues before the court during that term. To identify the 10 to 12 most important, publicly salient cases in each term, we either consulted directly with Supreme Court experts or read expert and journalistic commentary. For example, in June of 2020, the court’s docket included *Bostock v. Clayton County*, which questioned whether employers could fire workers based on their sexual orientation—a case of significant public salience that appeared on many “cases to watch” lists. We found that 83% of respondents (and 75% of Republican respondents) said this form of discrimination should be illegal. We then compared responses to the court’s eventual ruling holding that firing workers for being gay was indeed illegal under the Civil Rights Act. In the end, we asked about 32 cases in a similar fashion across the three surveys. (See *SI Appendix* for a full list of the cases and summary statistics for the court and respondents.)

We employ an ideal point model (13) that estimates the ideological position of each respondent based on their survey responses, each justice (and the court) based on their votes (and rulings) on all cases in a given term, and the perceived ideological position of the court held by each respondent based on their expectations about the court’s rulings in the surveyed cases. The unique pairing of our survey design and statistical approach allows us to estimate all of these quantities on a comparable scale within each year studied, enabling the direct comparison of the public’s preferences and the court’s.

The scale on which we measure ideology is constructed so that in each survey wave, the mean and SD of respondents’ estimated ideologies are zero and one, respectively, with lower (higher) values representing more liberal (conservative) ideologies. Accordingly, the scale corresponds to how many SDs above or below the average American a given ideological position is, which allows for easier interpretation of estimated positions. These ideology estimates are strongly associated with respondents’ own self-reported ideological identifications in the expected direction, and this association is relatively consistent across survey waves (*SI Appendix*, Fig. S1).

Note that we do not ex ante specify the directionality of each case in the ideal point model, but this is instead inferred by the model based on the vote data. Indeed, for the purposes of locating the court and the public in ideological space, our method does not require us to code any positions as liberal or conservative. Below we do refer to a conservative position as the one that is more supported by Republican respondents than by Democratic respondents (and vice versa for liberal positions), but this choice of language does not directly impact our ideology estimates.

It is possible that the ideological dimension structuring the votes of Supreme Court justices is different in some way from the ideological dimension structuring the views of ordinary Americans on these cases. Because we have so many more respondents (between 1,500 and 2,258 per survey) than justices in the data, the dimension we estimate is structured almost exclusively based on the views of these ordinary Americans. This fits well with the aim of our study since we are primarily concerned with the court’s behavior in comparison to the views of the public. Although previous work (14, 15) has proposed several methods for estimating latent constructs with multiple groups, we do not follow those approaches here because we have too few groups and, in the case of the court, too few members per group.

It is also possible that choosing different cases to survey the American public about would yield different estimates of justices’ and the court’s position relative to the public. As shown in *SI Appendix*, Figs. S3, S4 and S5, however, dropping any single case from any of our surveys does not meaningfully affect these ideology estimates. Further, *SI Appendix*, Fig. S2, shows that estimated ideological positions are similar overall when using only data on cases included in our survey (dropping other cases heard by the court each term), although this unsurprisingly results in a reduction in the precision of estimates, particularly for justices and the court. Although not dispositive, this suggests that our results are robust to changes in case selection.

Details on the statistical procedures for estimating the ideological positions of the court and public can be found in *SI Appendix*. Estimated ideological positions for each justice as well as the court and the public are presented in *SI Appendix*, Tables S1–S3.

### The Court’s Ideological Positioning Relative to the Public.

Our three surveys span 12 y and capture important political events, including changes in the identity of the court’s median justice. The median justice shifted from Anthony Kennedy to Roberts in October 2018 (following the retirement of Kennedy) and then from Roberts to Kavanaugh in November 2020 (following the death of Ruth Bader Ginsburg). To better understand partisan patterns, we disaggregate respondents into self-identified Democrats and Republicans. Following standard practice (16, 17), we pool respondents who lean toward one party with strong and not-strong partisans.

**Fig. 1.**
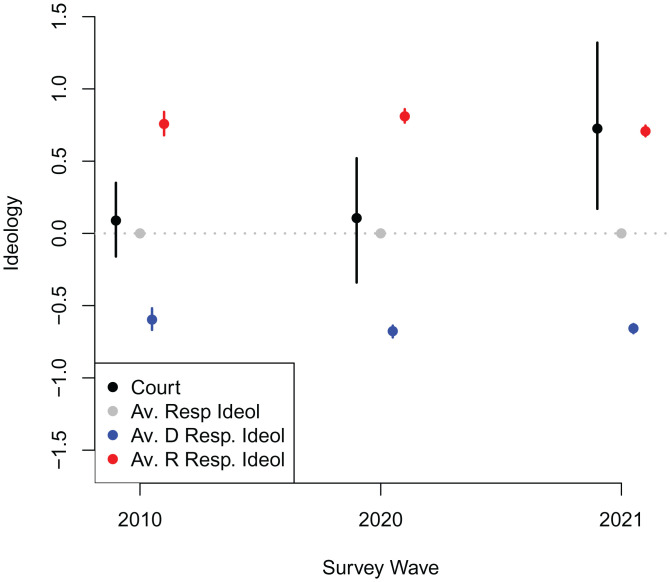
Court’s actual ideological positioning relative to the public’s. Black points show estimated ideology for Supreme Court (estimated based on majority position on each case included as a separate voter) in each year. Gray points show average estimated ideology for all respondents (note that the scale has been defined such that this is equal to zero in every year). Red (blue) points show average estimated ideology for Republican (Democratic) respondents. Vertical bars indicate 95% credible intervals for each estimate.

In Fig. 1, we show the ideological positioning of the court relative to the public and how both change over time. The court’s position, which can also be thought of as the position of the court’s majority, is estimated by including the court as a whole as a separate voter in the data, coding the court’s vote as the majority position in each case (i.e., the winning side). This allows us to estimate not only individual justices’ ideological positions but also the position of the court as an overall decision maker. Although there are theoretical reasons to expect majority decisions to move policy to the median voter’s position (18, 19), we do not impose this restriction in the ideal point model. The estimated court position, however, is quite close to the position of the justice estimated to be the median in all years we analyze (*SI Appendix*, Tables S1–S3).

In 2010, with Kennedy as the median, the court’s rulings put it in an ideological middle ground roughly halfway between Republicans and Democrats. In fact, the estimated ideological position of the court with Kennedy as the median falls almost exactly at the position of the average American. Somewhat surprisingly, this is also the case in 2020, after Roberts replaced Kennedy as median. Although this change was widely expected to produce a rightward shift in the court’s decisions, Roberts cast moderate votes on issues such as gay and transgender rights, as evidenced by the *Bostock* case mentioned above. His siding with liberal majorities across several issues, including health care and immigration, was noted by many in the media at this time (20). Hence, for the 2010 and 2020 waves, the court’s position was quite close to the average American despite the median justice being appointed by a Republican president in both years.

In 2021, however, with Amy Coney Barrett having replaced the liberal justice Ginsburg and the corresponding shift in the court’s median justice from Roberts to Kavanaugh, the court moved from a 5–4 conservative majority to a 6–3 conservative supermajority. This resulted in the court taking a sharp ideological shift to the right. Indeed, by the time of our 2021 survey the following April, the court is estimated to be significantly more conservative than the average American, falling close to the position of the average Republican.

To summarize, there was no meaningful change in the court’s ideological position relative to that of the general public, and relative to those of Republicans and Democrats, between 2010 and 2020, despite Roberts replacing Kennedy as the court’s median voter. There was, however, a sharp shift when Kavanaugh replaced Roberts as the court’s median in 2021, with the court moving away from the general public to correspond almost exactly to the ideological position of the average Republican voter.

### Expectations of the Court over Time.

We also asked respondents how they expected the court to rule in each of the 32 cases included in our surveys. For example, in the *Bostock* case, 83% of respondents (and 75% of Republicans) thought that it should be illegal to fire people based on their sexual orientation, but slightly fewer—79% of respondents (and 69% of Republicans)—actually thought the court would rule in that direction.

We use these questions to analyze how people’s expectations of the court have shifted over time and then compare these to how the court actually decided cases. This enables us to explore how accurately respondents perceive the court’s ideology and whether there may be a mismatch between expectations and reality.

**Fig. 2.**
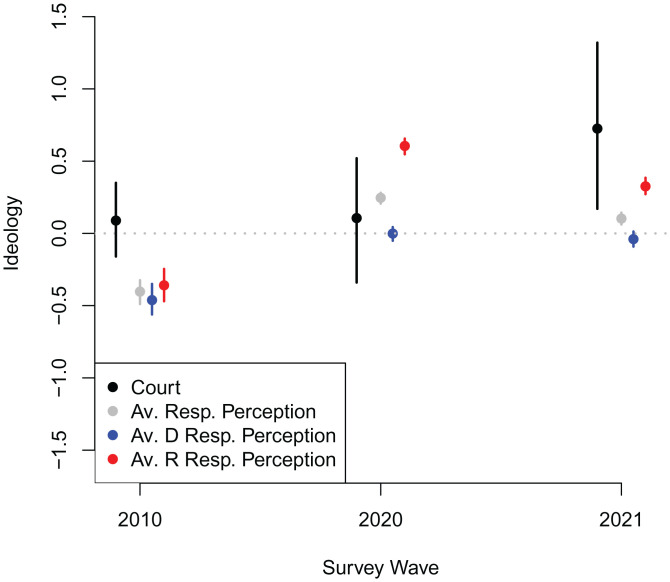
Expectations of how the court would rule relative to the court’s actual rulings. Black points show estimated ideology for Supreme court (estimated based on majority position on each case included as a separate voter) in each year. Gray points show average perception of the court by respondents in each year. Red (blue) points show average perception of the court by Republican (Democratic) respondents in each year. Vertical bars indicate 95% credible intervals for each estimate.

Fig. 2 shows these results, as well as the court’s actual ideological position based on the rulings. Estimated expectations for the groups shown in Fig. 2 are presented in *SI Appendix*, Tables S1–S3, along with uncertainty measures. Although a lot changed in US politics between 2010 and 2021, the results are revealing. First, in 2010, all groups (Republicans and Democrats, as well as the public as a whole) expected the court to be more liberal than it actually was. This suggests that Republican rhetoric about liberal activist courts perhaps shaped these perceptions (21) or perhaps that the legacy of the liberal Warren Court, at least in people’s minds, was still intact (22). Second, by 2020—after Roberts replaced the more moderate Kennedy as the median justice—all groups moved their expectations about the court rightward, but Republicans did so much more aggressively, perceiving the court as more conservative than its eventual rulings. Democrats’ perceptions were quite accurate.

Last, by the time of the 2021 survey, Kavanaugh replaced Roberts as the median, and a 6–3 conservative supermajority pushed the court’s rulings more to the right. However, Democrats in the 2021 survey wave had yet to fully update on this, still pegging their expectations of the court at 2020 levels, when Roberts was the median justice (ref. 10, chap. 5). As for Republicans, they actually backtracked slightly on their 2020 expectations of the court as an extremely right-leaning institution, putting their 2021 expectations of the court much closer to where the 2020 court actually landed.

As shown in *SI Appendix*, the overall patterns are fairly consistent across individual cases, but there are some notable topics that shed light on the trends between 2020 and 2021. In 2020, respondents accurately predicted that the court would take the liberal position on LGBT rights, whereas in 2021, respondents expected the court to be more liberal on criminal justice issues than it actually was.

These patterns suggest three takeaways: 1) in 2010 and 2021, respondents expected the court to be more liberal than it actually was; 2) although much changed in the time period involved, changes in the court’s composition—such as the changes between 2010 and 2020—are correlated with the shifts we observe; and 3) despite this, partisans in our data respond in different ways, with Democrats being particularly likely to consistently underestimate the court’s conservative lean.

### Attitudes toward Proposed Changes the Court.

Last, we explore how the growing ideological divergence between the public and the court maps onto attitudes on proposed institutional changes to the Supreme Court, a topic that has risen in salience since the 2016 US presidential election. We included two questions on the 2021 survey on 1) support for term limits for justices and 2) support for expanding the number of justices on the court.

Court expansion is not popular, with just under one-third of respondents giving a supportive response. Term limits, on the other hand, are fairly popular, with a slight majority of respondents supporting the proposal. (For both of these questions, slightly over a quarter of respondents said they neither agree nor disagree with the proposal, with the remainder giving an unsupportive response.) Democrats are more supportive of both measures than are Republicans, but the partisan difference in support is much larger for expanding the size of the court (51 and 16%) than for term limits (67 and 43%), and this difference is statistically significant (*P* < 0.001).

These baseline partisan differences, however, may mask associations between these views and how people perceive the court’s ideology in relation to their own. We therefore also examine the association between perceived ideological distance from the court and opinion on these issues (7). Specifically, we calculate each respondent’s perceived ideological position relative to the court as equal to their estimated perception of the court minus their own estimated ideological position. This captures how much more conservative (or liberal, for negative values) each respondent sees the court as being as compared to their own ideological position.

**Fig. 3.**
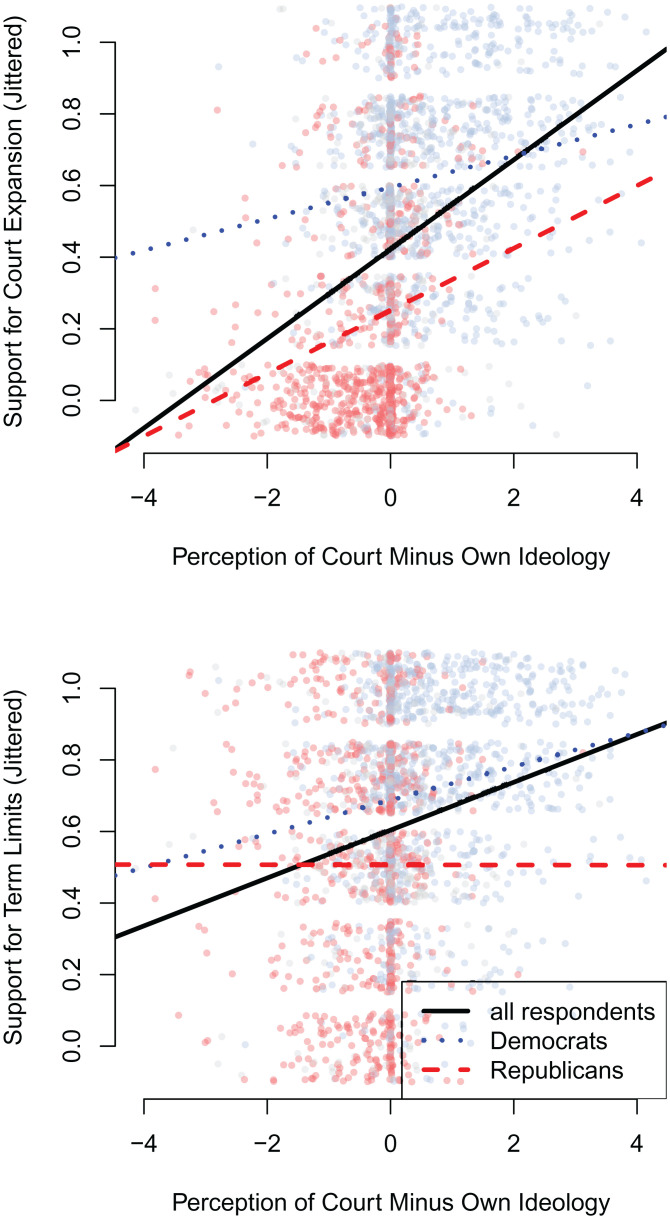
Predicting support for court expansion (*Top*) and term limits (*Bottom*) with respondent perceptions of court position relative to their own positions. Red, blue, and gray points indicate Republican, Democratic, and independent respondents, respectively. Black lines show linear regression estimates using all respondents, while red (blue) lines show linear regression estimates using only Republican (Democratic) respondents.

Fig. 3 plots each respondent’s perceived location relative to the court against their support for court expansion (Fig. 3, *Top*) and term limits (Fig. 3, *Bottom*). Responses to the court institutional change questions are rescaled so that 0 indicates strong opposition and 1 indicates strong support, and the responses on the four-point scale are jittered in the figure to avoid overplotting.

Although respondents are roughly evenly split between seeing the court as being more liberal (48%) versus more conservative (52%) than themselves, the average perceptual distance is 0.1, meaning that on average, respondents see the court as being slightly more conservative than they are. Overall, however, most respondents perceive the court as being fairly close to their own position. As seen in Fig. 1, this is a misperception: the court’s actual position is more conservative than the average respondent in 2021, the survey wave in which we asked about court expansion and term limits.

We also estimate linear regressions predicting support for each of the two institutional changes with these relative perceptions of the court. The black lines in each pane of Fig. 3 show these predicted relationships. In both cases, as respondents view the court as farther to their right, they become more supportive of the proposed changes to the court. This relationship is steeper when predicting support for Court expansion (β=0.12, *P* < 0.001) than when predicting support for term limits (β=0.07, *P* < 0.001). Although both institutional changes are supported by the majority of respondents who perceive the court as much more conservative than themselves, court expansion has much lower levels of support among other respondents.

We also examine these relationships separately among Democrats and Republicans, as shown by the blue dotted lines and red dashed lines, respectively, in each pane of Fig. 3. Comparing the two groups reveals that partisanship has a relatively small relationship to views on term limits, with Democrats being only slightly more supportive than Republicans who perceive themselves similarly relative to the court. These partisan differences, however, are larger when considering views on court expansion. For example, between a Democrat and a Republican who both perceive the court as having the same ideology as themselves, the Democrat would be predicted to give a response 0.22 units higher on support for expansion. (Recall this question is scaled to vary between 0 and 1.) *SI Appendix*, Fig. S1, presents loess fits that are overall similar to the linear regression results and suggest that (signed) distance between perception of the court and one’s own ideology, rather than absolute distance, is a more appropriate predictor in these models.

As shown in Fig. 2, many members of the public, particularly Democrats, misperceive the court’s ideology through 2021, underestimating its conservative nature. Since perceived ideological distance correlates with increased support for institutional change, this misperception matters. If people, particularly Democrats, actually knew the court’s conservative positioning with accuracy, they would likely be more supportive of making institutional changes to the court. To provide insight on this possible change, we use predicted values from the regressions that underlie Fig. 3. For example, in 2021, if the average American’s perception of the court changed from 0.10 to the actual estimated position of 0.73—that is, if they accurately perceived the conservative nature of the Court—then the regressions in Fig. 3 for all respondents would predict an increase of 0.04 and 0.08 in support for term limits and court expansion, respectively. Moving forward, it seems possible that these individuals will update their perceptions of the court’s ideology.

## Discussion

This paper makes several contributions. First, we use data taken at various points over a decade that ask people about the actual issues before the court. This enables us to jointly estimate and track the public’s preferences and the court’s rulings in a way that was previously not possible. We use these data to document the empirical fact that the court’s rulings have shifted over time vis à vis the public, putting the court in a much more conservative position since the appointment of Barrett.

This finding speaks to important discussions about how to best characterize the court’s ideology. Existing estimates of judicial ideology, such as Martin–Quinn scores (11), rely only on the justices’ votes to estimate judicial ideology and therefore do not allow for the comparison of the court’s position relative to the American public. By focusing on the publicly salient cases heard by the court in a given term, we are able to estimate the court’s position relative to the public’s views on these meaningful issues, allowing us to compare the court’s decision making over time to an important benchmark, the US public.

Second, we document that people’s expectations about how the court will rule fluctuate remarkably, showing correspondence with the changing composition of the court as well as to its rulings. Although the time period involved saw many political shifts, our findings show that as the court has moved to the right, so have the perceptions of what the court will do. We also note that many people—especially Democrats—currently perceive the court’s ideology incorrectly, with many believing it to be more liberal than it actually is. We leave it to future research to explore why this might be and whether these patterns endure, especially if the court’s more conservative rulings attract additional attention. It is also possible the court’s positioning will change and swing back toward the middle.

Third, we bring these analyses together to examine what these patterns mean for the future of the court’s institutional structure. We find that greater distance between how people perceive the court and their own ideology predicts their potential support for court-curbing measures. This in turn suggests an important takeaway: because many people underestimate the court’s conservative leaning, this may be leading to less support for institutional changes than would be the case if they had an accurate understanding.

By conducting three surveys as part of a decade-long data collection effort, our goal has been to provide empirical regularities. These not only shed light on the development of the court as an institution but also generate important research questions going forward.

## Materials and Methods

### Survey Context and Design.

We conducted three nationally representative surveys, each asking respondents’ opinions on the key issues in prominent cases in the Supreme Court’s docket and also asking respondents how they expected the court to rule in each case. These surveys were conducted in April 2010 (n=1500), April 2020 (*n* = 2000), and April 2021 (*n* = 2158). The surveys were timed to correspond to changes in the identity of the median justice. Information on the cases asked about in the surveys in *SI Appendix*. This research received institutional review board (IRB) approval from The University of Texas at Austin (no. IRB2020-03-0046), Stanford University (nos. IRB55200 and IRB-18544), and Harvard University (nos. IRB21-0341 and IRB20-0407). Respondents were told that they could stop participating at any time and informed of the survey’s basic content and length before agreeing to participate. Prior to starting the survey, respondents were shown an information screen that communicated participant rights, risks and benefits, and independent IRB contact information.

In between these survey waves, the court’s composition and also its docket and rulings changed dramatically, which is key for the questions we investigate. At the time of the 2010 survey, there were five conservatives and four liberals, with the relatively moderate Anthony Kennedy operating as the court’s median. By the time of the 2020 survey, there were also five conservatives and four liberals, but with Brett Kavanaugh replacing Kennedy, the more-conservative John Roberts took over the median’s role. By the time of the 2021 survey, with Amy Coney Barrett replacing the liberal Ruth Bader Ginsburg, the Court’s median again shifted to the right, this time to Kavanaugh. Thus, the three survey waves meaningfully capture discrete shifts in the Court’s composition and their rulings. This presents a unique opportunity to examine correspondence with public opinion at these key moments of Supreme Court change. A decade-long longitudinal effort is needed to capture pivotal changes in the court’s composition, which changes very slowly over time.

Each survey was administered online by YouGov using a representative sample of American adults recruited as part of their main panel study. Using sample matching procedures, YouGov matches respondents to representative benchmark datasets such as the American Community Survey, the Current Population Survey, the Pew US Religious Landscape Survey, and voter files (23). YouGov draws a random sample of respondents from these data sources to create a target sample. It then matches individuals from its opt-in Internet survey panel via perfect replacement such that the survey sample is equivalent to the target sample. Sample matching has been shown to perform extremely well; studies that have conducted concurrent surveys comparing YouGov against probability samples demonstrate extremely similar results across sampling methods (23, 24). This includes not only means and distributions of variables but also relationships among survey variables and similarities to real-world benchmarks. Descriptive statistics of the three samples can be found in *SI Appendix*, Table S4. All analyses apply poststratification weights provided by YouGov. Applying these weights ensures that the survey sample matches the target sample given nonresponse.

### Ideal Point Estimation.

Each survey wave included a series of questions asking respondents about 1) their views about several cases heard by the court in that term and also 2) how they expected the court to actually rule on the cases. The intent behind these questions was to ask respondents about the most prominent, salient cases that would be the most likely to impact public policy and, thus, also shape people’s perceptions of the court. For the 2010 survey wave, cases were selected by examining lists of notable cases that had been recently decided by the court. For the 2019 to 2020 term, we use a modified list taken from an overview of important Supreme Court cases that appeared contemporaneously in *USA Today* (https://www.usatoday.com/pages/interactives/news/supreme-court-decisions-2019-2020/). For the 2020 to 2021 term, we worked with the Supreme Court reporter at the *New York Times* to develop the list of cases. For the 2020 and 2021 waves, respondents were interviewed prior to the court ruling on the cases. For the 2010 wave, the survey was fielded after the cases had already been decided. As discussed in ref. 25, there did not appear to be significant differences in public opinion or the relationship between ideological position and views of the court when separately analyzing high-salience and low-salience cases in the 2010 survey.

Of course, asking about complex cases in a short survey question is difficult. Thus, our objective was not to ask respondents about cases’ jurisprudence issues, which would be meaningless to the vast majority of respondents, but, instead, to focus on the policy implications of judicial rulings. The list of cases and question wordings is presented in *SI Appendix*. Descriptive statistics on respondents’ views of the cases and expectations of the court can be found in *SI Appendix*, Tables S5 and S6.

Our approach to estimating justices’ ideologies as well as public opinion and public perceptions of the Court follows the approach used by ref. 13. Letting *y_ij_* be 1 if actor *i* supports the Supreme Court’s majority position on case *j* (where actor can refer to a justice, a survey respondent, or a survey respondent’s expectation about the court’s ruling), the model assumes that $P(y_{ij}=1)=\Phi (\beta_{j}x_{i}-\alpha_{j})$, where *β_j_* is the discrimination parameter for case *j* indicating how an individual’s ideological position *x_i_* predicts the likelihood of supporting the Court’s majority position on that case, and *α_j_* is the difficulty parameter, indicating how much baseline support there is for the majority position in the case. Further details about this approach, including estimation, can be found in *SI Appendix*.

Because survey respondents are asked their opinions on the same issues decided by the court, we can estimate justices’ ideologies on the same scale as public opinion by treating survey respondents as if they had each cast a vote on each of the surveyed cases. Additionally, we can treat each respondent’s perception of the court as driving their expectations (guesses) about how the court will decide each surveyed case.

We create a vote matrix stacking justices’ votes on all cases in a given term, respondents’ positions on the surveyed cases, and respondents’ guesses about the court’s decision in each surveyed case. This means that rows for respondents, and also for respondent perceptions, will have mostly missing values since we surveyed only a fraction of the cases in each term. The ideal point model then estimates the ideological position of each justice, of the court (included as a separate voter siding with the majority on every case), of each survey respondent, and of each survey respondent’s expectation of the court, all on the same single dimension. We transform this scale so that the average estimated ideology of survey respondents is zero and the SD of estimated respondent ideology is 1. Lower (higher) values on this scale represent more liberal (conservative) positions.

### Measures.

From the three surveys, we calculated several key quantities.

#### Perceived distance from court.

In addition to asking people where they stood on the cases, we also asked them their expectations about how the court would decide, following the substantive implications of ref. 7, using these expectations to create an ideal point measure for perceptions of the court. Then, we then take the difference between people’s actual ideal points and the perceived ideal points. This allows us to assess people’s perceived cardinal distance from the court on the issues it decides.

#### Proposed changes to the court.

We asked respondents the following: “The US Supreme Court has nine members. Some people believe that Congress should expand the size of the Supreme Court, allowing the current president to appoint one or more new Justices. Do you agree or disagree that the size of the Supreme Court should be increased?” (response options were “strongly agree,” “agree,” “neither agree nor disagree,” “disagree,” and “strongly disagree”). Respondents were also asked the following: “US Supreme Court Justices currently serve life terms. Some people think that, instead, Supreme Court Justices should be limited to 18-year terms. Do you agree or disagree that there should be such term limits for Supreme Court Justices?” (response options were “strongly agree,” “agree,” “neither agree nor disagree,” “disagree,” and “strongly disagree”).

#### Party identification.

For all three survey waves, respondents were first asked “Generally speaking, do you usually think of yourself as a …” (response options were “Republican,” “Democrat,” “Independent,” and “Other”). Republicans and Democrats were then asked “Would you call yourself a strong [Republican/Democrat] or a not very strong [Republican/Democrat]?” Nonpartisans were asked “Do you think of yourself as closer to the Republican Party or to the Democratic Party?” Leaners were pooled in with partisans in analyses examining effects among Republicans and Democrats.

## Supplementary Material

Supplementary File

## Data Availability

Replication materials for the results in this article and the *SI Appendix* are available at Harvard Dataverse (https://doi.org/10.7910/DVN/5J8R2J) (26).
