# Supplementary material for: A decade-long longitudinal survey shows that the Supreme Court is now much more conservative than the public
Source: Proc Natl Acad Sci U S A. 2022 Jun 6;119(24):e2120284119. doi: 10.1073/pnas.2120284119 (PMC9214517; doi:10.1073/pnas.2120284119)
Supplement: Supplementary File [file pnas.2120284119.sapp.pdf]

Supplementary Information for

A decade-long longitudinal survey shows that the Supreme Court is now much more conservative than the public

Stephen Jessee, Neil Malhotra, and Maya Sen

Corresponding Author Stephen Jessee.
sjessee@utexas.edu

This PDF file includes:

Supplementary text
Figs. S1 to S7
Tables S1 to S6
SI References

Supporting Information Text

Case Questions

The survey questions for each of the 32 Supreme Court cases asked about over the three waves is found below. Respondents were randomly assigned to read one of two versions of each question, with the response options flipped (both in the question stem and the answer choices). After providing their own opinion on the case, respondents were then asked: "What do you think the Supreme Court would decide if they were asked to rule on this issue?" with the same response options.

2021 Wave

Case #1. *Fulton v. City of Philadelphia PA*

There are some religiously affiliated foster agencies that refuse to place foster children with same-sex couples.

Some people think that governments can prohibit such agencies from participating in the foster care systems they operate unless the agencies allow children to be placed with same-sex couples. Other people think that doing so would violate the agencies' First Amendment rights to religious freedom.

What do you think?

Prohibiting religious agencies from participating in the foster care system unless they allow children to be placed with same-sex couples DOES NOT VIOLATE agencies' First Amendment rights to religious freedom

Prohibiting religious agencies from participating in the foster care system unless they allow children to be placed with same-sex couples DOES VIOLATE agencies' First Amendment rights to religious freedom

Case #2. *Brnovich v. Democratic National Committee I*

In Arizona, if a voter arrives at a polling place and is not listed on the voter roll for that precinct, the voter may still cast a provisional ballot. After election day, Arizona election officials review all provisional ballots to determine the voter's identity and address. If officials determine that the voter voted outside of their precinct, the ballot is discarded in its entirety, even if the voter was eligible to vote in most of the races on the ballot. Some people believe that discarding entire ballots in this manner is unlawful. Other people believe that it is lawful.

What do you think?

Discarding entire ballots from voters who voted outside of their precinct IS UNLAWFUL

Discarding entire ballots from voters who voted outside of their precinct IS LAWFUL

Case #3. *Brnovich v. Democratic National Committee II*

Arizona offers in-person voting at a precinct or vote center either on election day or during an early-vote period. Many voters – particularly racial minorities – who vote early rely on another person to collect and drop off voted ballots. However, the Arizona legislature made it illegal to collect and deliver another person's ballot. Some people think that voters should be able to rely on another person or third party to collect and drop off ballots. Other people think that states can forbid this.

What do you think?

Voters SHOULD BE ABLE to rely on another person or third party to collect and drop off ballots

STATES CAN FORBID voters from relying on another person or third party to collect and drop off ballots

Case #4. *Americans for Prosperity Foundation v. Becerra*

To detect possible fraud, the attorney general of California requires private nonprofit organizations to report the names and addresses of their major donors to the state, which keeps this information confidential. Some people think that this violates nonprofit organizations' First Amendment rights to free association because it might deter people from financially supporting them. Other people do not think that this violates nonprofit organizations' First Amendment rights to free association.

What do you think?

Requiring nonprofit organizations to report information to the state about their major donors VIOLATES their First Amendment rights

Requiring nonprofit organizations to report information to the state about their major donors DOES NOT VIOLATE their First Amendment rights

Case #5: *Mahanoy Area School District v. B.L.*

Some people think that public school officials can punish students for things they say or write off campus, including on social media, without violating students' First Amendment rights to free speech. Other people think that such punishments violate students' First Amendment rights to free speech.

What do you think?

Public school officials CAN punish students for things they say or write off campus

Public school officials CANNOT punish students for things they say or write off campus

Case #6: *Jones v. Mississippi*

There are states that reserve the ability to sentence juvenile criminal defendants to life sentences without the possibility of any parole. Some people think that such juvenile defendants must be found to be incorrigible – or impossible of being reformed – before being sentenced to life without parole. Other people think that juveniles can be sentenced to life sentences without parole without states having to make such a determination.

What do you think?

Juvenile defendants MUST BE FOUND to be incorrigible before being sentenced to a life sentence without parole

Juvenile defendants NEED NOT BE FOUND to be incorrigible before being sentenced to a life sentence without parole

Case #7: *Van Buren v. United States*

Law enforcement officers have access to several government databases to use for investigations and other law enforcement activities. Some people believe that using government databases for uses not explicitly authorized should be punished, even if the user had lawful access to the database. Others believe that using government databases for other uses not explicitly authorized should not be punished, so long as the user had lawful access to the database.

What do you think?

Using government databases for uses not explicitly authorized SHOULD BE PUNISHED

Using government databases for uses not explicitly authorized SHOULD NOT BE PUNISHED

Case #8: *National Collegiate Athletic Association v. Alston*

The National Collegiate Athletic Association (NCAA) strictly limits colleges and universities from providing paid compensation to college athletes. Some people think the NCAA's strict limits on paid compensation for college athletes in this manner is an unlawful form of coordination against athletes. Others disagree and think that the NCAA should be able to strictly limit colleges and universities from providing paid compensation to college athletes.

What do you think?

The NCAA SHOULD NOT BE ABLE TO strictly limit paid compensation to college athletes.

The NCAA SHOULD BE ABLE TO strictly limit paid compensation to college athletes. state

Case #9: *Roman Catholic Diocese of Brooklyn v. Cuomo*

Many states have prohibited large in-person gatherings due to the COVID-19 pandemic. Some people think that states cannot prohibit in-person religious gatherings because of the First Amendment right to free exercise of religion. Other people think that states can prohibit in-person religious gatherings.

What do you think?

States CANNOT prohibit in-person religious gatherings because of the First Amendment right to free exercise of religion

States CAN prohibit in-person religious gatherings despite the First Amendment right to free exercise of religion

Case #10: *Cedar Point Nursery v. Hassid* California law requires that employers allow union representatives to enter a company's private property to meet with employees and solicit support for labor organizing. Some people believe that this is akin to the government taking companies' private property without compensation. Other people argue that the law is acceptable, and is not the government taking companies' private property without compensation.

What do you think?

States CANNOT require that employers allow union representatives to enter a company's private property

States CAN require that employers allow union representatives to enter a company's private property

Case #11: *Collins v. Mnuchin* Some federal government agencies – such as the Federal Housing Finance Agency – are headed by a single director who may be removed from office by the president only for a specific cause rather than for any reason the president wishes. Some people think that this leadership structure is unconstitutional because it infringes on the president's authority over the executive branch, including such federal agencies. Other people disagree and think that this leadership structure is not unconstitutional because it does not infringe on the president's authority over the executive branch.

What do you think?

Limiting the president to only firing agency heads for a specific reason INFRINGES on the president's authority

Limiting the president to only firing agency heads for a specific reason DOES NOT INFRINGE on the president's authority

Case #12: *Lange v. California*

Police officers sometimes pursue people who flee in cars and who refuse to pull over. Some people think that a police officer should be able to enter someone's home without a warrant if they are in pursuit of them when that person enters their home. Other people think that it is unconstitutional for police to enter someone's home without a warrant except when there is a genuine emergency.

What do you think?

Police SHOULD be able to enter someone's house without a warrant if they are in pursuit of them when that person enters their home

Police SHOULD NOT be able to enter someone's house without a warrant if they are in pursuit of them when that person enters their home

2020 Wave

Case #1. *Bostock v. Clayton County, Georgia*

Some people believe that it should be illegal for employees to be fired based on their sexual orientation because it is discrimination on the basis of sex. Other people think that it should be legal because it is not discrimination on the basis of sex.

What do you think?

It should be ILLEGAL for employees to be fired based on their sexual orientation

It should be LEGAL for employers to fire people based on their sexual orientation

Case #2. *R.G. & G.R. Harris Funeral Homes Inc. v. Equal Employment Opportunity Commission*

Some people believe that it should be illegal for employees to be fired for being transgender because it is discrimination on the basis of sex. Other people think that it should be legal because it is not discrimination on the basis of sex.

What do you think?

It should be ILLEGAL for employees to be fired for being transgender.

It should be LEGAL for employees to be fired for being transgender.

Case #3. *Department of Homeland Security v. Regents of the University of California*

Deferred Action for Childhood Arrivals (DACA) was created by President Obama to protect undocumented immigrants who have lived in the U.S. since childhood from deportation. President Trump wants the Department of Homeland Security to end DACA.

What do you think?

DACA should remain

DACA should be ended

Case #4. *Espinoza v. Montana Department of Revenue*

The state of Montana has banned students from using taxpayer-subsidized scholarships to attend religious schools. Some people think this rule is an acceptable restriction. Other people think this rule violates people's constitutional rights.

What do you think?

States SHOULD be allowed to ban students from using taxpayer-subsidized scholarships to attend religious schools

States SHOULD NOT be allowed to ban students from using taxpayer-subsidized scholarships to attend religious schools

Case #5. *Little Sisters of the Poor Saints Peter and Paul Home v. Pennsylvania*

The Affordable Care Act requires that health insurance plans for women include coverage for contraceptives (birth control), but the Trump administration recently passed regulations that greatly expanded exceptions to this mandate to include exemptions on the basis of religious or "moral" objections. Some people think that employers should not be forced to cover contraceptives if they express either a religious or a "moral" objection. Other people think that these employers should be forced to cover contraceptives.

What do you think?

Employers SHOULD NOT be forced to cover contraceptives

Employers SHOULD be forced to cover contraceptives

Case #6. *June Medical Services, LLC v. Russo*

Louisiana passed a law requiring abortion providers to be able to send patients to nearby hospitals, a practice known as "admitting privileges." This law would mean that all abortion providers in the state except for one would be forced to close. Some people believe that Louisiana's law violates women's constitutional rights. Other people believe that the law does not violate women's constitutional rights.

What do you think?

States requiring abortion providers to have admitting privileges DOES violate women's constitutional rights

States requiring abortion providers to have admitting privileges DOES NOT violate women's constitutional rights

Case #7: *Trump v. Deutsche Bank AG* and *Trump v. Mazars USA, LLP*

A Congressional committee has requested records related to President Trump's taxes and finances from his activities prior to becoming president from his accounting firm and other companies. Some people believe that a president should be able to block such companies from turning over his financial records to Congressional committees. Other people believe that the companies must comply with the Congressional committee's request.

What do you think?

A president SHOULD be able to block turning over his financial records to Congress

A president SHOULD NOT be able to block turning over his financial records to Congress

Case #8: *Trump v. Vance*

New York state prosecutors are conducting a criminal investigation of President Trump. They have requested financial records related to President Trump's taxes and finances from his activities prior to becoming president from his accounting firm and other companies. Some people believe that New York state prosecutors have the right to obtain a president's tax records. Others believe that a president does not have to turn over his tax records to state prosecutors.

What do you think?

New York state prosecutors have the right to obtain a president's tax records

A president does not have to turn over his tax records to state prosecutors

Case #9: *Seila Law, LLC v. CFPB*

In 2010, Congress established the Consumer Financial Protection Bureau (CFPB) as an independent consumer protection agency. In doing, so Congress severely limited the President's ability to remove the agency's director. Some people think the structure of the CFPB is appropriate. Others disagree and believe that this gave the CFPB too much independent power, since it is very difficult for the President to remove the agency's director.

What do you think?

The structure of the CFPB is appropriate

The CFPB has too independent power

Case #10: *Chiafalo v. Washington* and *Colorado Department of State v. Baca*

In the U.S., the President is chosen by the Electoral College, comprised of "electors" from all 50 states and the District of Columbia. Some people think that states should be able to require Electoral College electors to vote for the person who won the majority of votes in the state and not some other person. However, some people think that electors should be able to vote for whomever they want.

What do you think?

States SHOULD be able to require their "electors" to vote for the candidate who won their state

States SHOULD NOT be able to require their "electors" to vote for the candidate who won their state

2010 Wave

Case #1: *U.S. v. Comstock*

Some people believe that the federal government should have the authority to place sex offenders in mental institutions involuntarily after their original prison sentences have ended. Other people believe that the federal government must release these people after their original prison sentences have ended.

What do you personally believe?

The federal government must release sex offenders after their prison sentences have ended,

The federal government should be able to involuntarily put sex offenders in mental institutions after their prison sentences have ended

Case #2: *McDonald v. Chicago*

Some people believe that state and local governments should have the ability to ban the possession of handguns. Other people believe that the right of individual citizens to possess handguns is protected under the Second Amendment.

What do you personally believe?

State and local government should be able to ban the possession of handguns

The right of individual citizens to possess handguns is protected under the Second Amendment

Case #3: *Salazar v. Buono*

Some people believe that religious symbols and statues such as crosses should be allowed to be placed on government-owned land. Other people believe that placing religious symbols on government-owned land represents a violation of the separation of church and state.

What do you personally believe?

Religious symbols should be allowed to be placed on public land.

Placing religious symbols on public land violates the separation of church and state.

Case #4: *Ricci v. DeStefano*

Some people believe that a city should be allowed to try to increase racial diversity by denying the promotion of white firefighters who passed a promotion test because no black firefighters passed the test. Other people believe that the city should be required to abide by the results of the promotion test and promote the white firefighters even if no black firefighters are promoted.

What do you personally believe?

The city should be allowed to deny the promotion of the white firefighters.

The city must abide by the results of the test and promote the white firefighters

Case #5: *Crawford v. Marion County*

Some people believe that states can require voters to provide photo identification at the polling place. Other people believe that requiring people to show photo identification violates voting rights.

What do you personally believe?

States can require voters to show photo identification,

Requiring voters to show photo identification violates voting rights

Case #6: *Citizens United v. FEC*

Some people believe that prohibiting corporations from contributing to political campaigns a violation of the right to free speech guaranteed by the First Amendment. Other people believe that this is a legitimate restriction that promotes more democratic elections.

What do you personally believe?

Prohibiting corporations from contributing to political campaigns is a violation of the right to free speech,

Prohibiting corporations from contributing to political campaigns is a legitimate restriction that promotes more democratic elections

Case #7: *Baze v. Rees*

Some people believe that lethal injection should be allowed to be used to execute convicted criminals on death row. Other people believe that using lethal injection is cruel and unusual punishment.

What do you personally believe?

Lethal injection should be allowed.

Lethal injection is cruel and unusual punishment.

Case #8: *Parents Involved v. Seattle*

Some people believe that school districts should be allowed to assign students to schools based on race in order to promote diversity and reduce segregation. Other people believe that race should not play a factor at all in assigning students to schools.

What do you personally believe?

School districts should be allowed to use race to promote diversity

Race should not be used to assign students to school

Case #9: *Gonzales v. Carhart*

Some people believe that the federal government should be allowed to ban partial birth abortions. Other people believe that banning this procedure violates women's rights.

What do you personally believe?

The federal government should be allowed to ban partial birth abortions.

Banning partial birth abortions violates women's rights.

Case #10: *Hamdan v. Rumsfeld*

Some people believe that the federal government should be allowed to conduct military trials of suspected terrorists in Guantanamo Bay. Other people believe that these individuals have the right to be tried in the American court system.

What do you personally believe?

The federal government should be allowed to conduct military trials of suspected terrorists.

Suspected terrorists have the right to be tried in the American court system

Ideology Estimation

Our approach to estimating justices' ideologies as well as public opinion and public perceptions of the Court follows the approach used by (1). Letting y_{ij} be 1 if actor i supports the Supreme Court's majority position on case j (where "actor" can refer to a justice, a survey respondent, or a survey respondent's expectation about the Court's ruling), the model assumes that $P(y_{ij} = 1) = \Phi(\beta_j x_i - \alpha_j)$ where β_j is the discrimination for case j indicating how an individual's ideological position x_i predicts the likelihood of supporting the Court's majority position on that case, and α_j is the difficulty parameter indicating how much baseline support there is for the majority position in the case.

The parameters of the model are estimated separately for each year in our data (2010, 2020 and 2021). We create a vote matrix stacking justices' votes on all cases in a given term, respondents' positions on the surveyed cases, and respondents' guesses about the Court's decision in each surveyed case. This means that rows for respondents, and also for respondent perceptions, will have mostly missing values since we surveyed only a fraction of the cases in each term.

Model fitting is done using Markov chain Monte Carlo (MCMC) methods employed in the `ideal` function from the `pscl` R package (2). Default priors are used (independent standard normal for each x_i and normal with mean zero and variance 25 for each of the β_j and α_j parameters). Initial values for the ideal points x_i are based on the proportion of positions taken on each case that are conservative, and initial values for α_j and β_j are obtained by running probit regressions predicting positions for each case using these x_i's. The sampler is run for 100,000 burn in iterations and then 400,000 subsequent iterations are run,

storing every 100th iteration for a total of 4,000 stored iterations. Standard convergence diagnostics suggested that the sampler converged.

We constrain each iteration of the sampler so that the mean and standard deviation of all ideal points are 0 and 1, respectively (this is implemented with the `normalize=TRUE` option in the `ideal` function). Finally, we transform the estimated ideal points so that the average estimated ideology of survey respondents is zero and the standard deviation of estimated respondent ideology is one. Lower (higher) values on this scale represent more liberal (conservative) positions. This is to allow for ease of interpretation of the resulting ideology scale.

The top sections of Tables S1, S2, and S3 show, for 2010, 2020 and 2021, the estimated ideological positions (posterior means for x_i) for each justice and for the Supreme Court as a whole, which is operationalized as a voter who supports the majority position in each case. To summarize the uncertainty in these estimates, we also present the lower and upper bounds of 95 percent highest posterior density regions (HPDs) for each quantity. These are essentially a Bayesian analogue of confidence intervals. The middle sections of these tables show estimates and HPDs for the average ideology of all respondents as well as among Democratic and among Republican respondents. The lower sections of these tables show estimates and HPDs for average perceptions of the Court among all respondents as well as among Democratic and among Republican respondents. These numbers correspond to those in Figures 1 and 2 in the main paper.

Table S1. 2010 Wave Ideology Estimates

	Estimate	HPD low	HPD high
Stevens	-1.87	-2.37	-1.40
Sotomayor	-1.39	-1.89	-0.94
Ginsburg	-1.25	-1.57	-0.94
Breyer	-1.13	-1.42	-0.82
Kennedy	0.10	-0.15	0.36
Roberts	0.82	0.50	1.16
Alito	0.95	0.60	1.33
Scalia	1.43	0.97	1.88
Thomas	2.44	1.70	3.30
Majority	0.09	-0.16	0.35
Average Ideology:			
All Respondents	0	0	0
Democrats	-0.60	-0.67	-0.52
Republicans	0.76	0.68	0.84
Average Perception of Court:			
All Respondents	-0.40	-0.49	-0.32
Democrats	-0.46	-0.56	-0.35
Republicans	-0.36	-0.47	-0.25

Table S2. 2020 Wave Ideology Estimates

	Estimate	HPD low	HPD high
Sotomayor	-1.61	-2.51	-0.83
Ginsburg	-1.27	-1.98	-0.61
Breyer	-1.06	-1.65	-0.49
Kagan	-1.05	-1.63	-0.49
Roberts	0.29	-0.15	0.72
Gorsuch	0.40	-0.08	0.89
Kavanaugh	0.43	-0.02	0.89
Alito	1.85	1.00	2.75
Thomas	2.55	1.42	3.65
CourtMajority	0.11	-0.34	0.52
Average Ideology:			
All Respondents	0	0	0
Democrats	-0.68	-0.72	-0.64
Republicans	0.81	0.77	0.86
Average Perception of Court:			
All Respondents	0.25	0.21	0.28
Democrats	-0.00	-0.05	0.04
Republicans	0.61	0.55	0.66

Table S3. 2021 Wave Ideology Estimates

	Estimate	HPD low	HPD high
Sotomayor	-2.49	-3.69	-1.34
Breyer	-1.34	-2.20	-0.54
Kagan	-1.00	-1.74	-0.27
Roberts	0.75	0.19	1.26
Kavanaugh	0.78	0.28	1.35
Barrett	1.20	0.61	1.83
Thomas	1.44	0.70	2.14
Gorsuch	1.51	0.83	2.23
Alito	2.13	1.19	3.12
CourtMajority	0.73	0.17	1.32
Average Perception of Court:			
All Respondents	0	0	0
Democrats	-0.66	-0.69	-0.63
Republicans	0.71	0.67	0.75
Average Perception of Court:			
All Respondents	0.10	0.06	0.14
Democrats	-0.04	-0.09	0.01
Republicans	0.33	0.27	0.39

Descriptive Statistics

	2021	2020	2010
**Gender**			
Male	48.2%	48.2%	48.1%
Female	51.8%	51.8%	51.9%
**Age**			
Under 30	20.2%	21.9%	18.9%
30-44	24.8%	25.4%	28.3%
45-64	33.7%	34.1%	35.7%
65+	21.3%	18.6%	17.1%
**Race**			
White	63.2%	64.3%	73.9%
Black	12.5%	12.3%	12.1%
Hispanic	15.5%	15.8%	6.3%
Asian	3.7%	3.6%	2.8%
Native American	0.4%	0.8%	0.9%
Middle Eastern	2.2%	1.4%	0.4%
Mixed	2.3%	1.7%	2.0%
Other	0.1%	0.1%	1.6%
**Education**			
High School or Less	38.9%	40.0%	43.6%
Some college	27.8%	30.8%	23.7%
College grad	21.3%	20.0%	24.7%
Postgrad	12.0%	9.3%	8.0%
**Partisanship**			
Republican	34.4%	33.7%	31.5%
Democrat	40.6%	41.0%	34.4%
Independent/Other	25.0%	25.3%	34.1%
**N**	2158	2000	1500

Table S4. Descriptive Statistics of Survey Samples

					% agreeing with decision			% correctly predicting Court decision		
Term	Survey Wave	Case	Topic	Court Decision	Full Sample	Reps	Dems	Full Sample	Reps	Dems
2020-2021	2021	Fulton v. City of Philadelphia PA	Gay Adoption	Conservative	52.2%	65.6%	38.9%	53.8%	56.1%	53.6%
2020-2021	2021	Brnovich v. Democratic National Committee I	Provisional Ballots	Conservative	49.1%	67.7%	33.9%	53.4%	59.3%	52.2%
2020-2021	2021	Brnovich v. Democratic National Committee II	Ballot Harvesting	Conservative	50.0%	75.2%	29.2%	56.3%	66.7%	49.7%
2020-2021	2021	Mahanoy Area School District v. B.L.	School Free Speech	Conservative	70.5%	80.2%	63.3%	67.0%	73.5%	64.3%
2020-2021	2021	Jones v. Mississippi	Juvenile Crime	Conservative	29.4%	36.0%	22.0%	34.9%	31.9%	36.9%
2020-2021	2021	Van Buren v. United States	Databases	Conservative	31.9%	31.8%	31.2%	37.3%	33.8%	39.9%
2020-2021	2021	National Collegiate Athletic Association v. Alston	NCAA Athletes	Liberal	49.9%	39.7%	58.1%	48.0%	41.1%	54.9%
2020-2021	2021	Cedar Point Nursery v. Hassid	Unions	Conservative	51.6%	70.6%	34.4%	55.4%	62.0%	52.4%
2020-2021	2021	Lange v. California	Warrants	Liberal	52.4%	41.2%	60.0%	49.8%	44.2%	53.8%
2020-2021	2021	Americans for Prosperity Foundation v. Becerra	Donors	Conservative	40.0%	56.1%	25.5%	48.0%	49.7%	46.6%
2019-2020	2021	Roman Catholic Diocese of Brooklyn v. Cuomo	COVID Restrictions	Conservative	53.6%	77.4%	29.0%	62.8%	70.0%	58.3%
2020-2021	2021	Collins v. Mnuchin	Federal Agencies	Conservative	45.5%	50.1%	39.9%	44.7%	45.9%	41.0%
2019-2020	2020	Espinoza v. Montana Department of Revenue	Scholarships	Conservative	63.1%	76.6%	52.3%	63.8%	67.5%	61.3%
2019-2020	2020	Trump v. Deutsche Bank AG	Trump Taxes (Congress)	Liberal	60.9%	30.9%	84.5%	51.9%	35.7%	61.5%
2019-2020	2020	Seila Law, LLC v. CFPB	CFPB	Conservative	43.6%	69.4%	20.8%	49.6%	65.3%	36.0%
2019-2020	2020	Bostock v. Clayton County, Georgia	Firing Gay Employees	Liberal	83.3%	74.6%	90.4%	81.3%	81.8%	80.7%
2019-2020	2020	R.G. & G.R. Harris Funeral Homes, Inc. v. EEOC	Firing Transgender Employees	Liberal	78.8%	68.6%	87.2%	76.8%	78.5%	75.4%
2019-2020	2020	DHS v. Regents of the University of California	DACA	Liberal	61.0%	30.4%	85.5%	54.3%	41.9%	63.8%
2019-2020	2020	June Medical Services, LLC v. Russo	Abortion	Liberal	56.9%	37.3%	73.6%	47.8%	39.5%	52.3%
2019-2020	2020	Trump v. Vance	Trump Taxes (State)	Liberal	61.3%	28.0%	85.5%	49.8%	30.9%	59.8%
2019-2020	2020	Chiafalo v. Washington	Electoral College	Liberal	61.4%	59.5%	65.0%	55.5%	55.1%	58.4%
2019-2020	2020	Little Sisters of the Poor v. Pennsylvania	Contraceptives	Conservative	52.7%	70.4%	33.3%	63.2%	68.8%	56.3%
2009-2010	2010	McDonald v. Chicago	Gun Control	Conservative	71.4%	92.8%	56.6%	76.9%	85.6%	74.9%
2008-2009	2010	Ricci v. DeStefano	Affirmative Action	Conservative	89.6%	95.7%	81.8%	76.3%	75.2%	75.2%
2006-2007	2010	Parents Involved v. Seattle	Race and Schools	Conservative	84.8%	98.1%	74.9%	63.8%	63.1%	62.3%
2005-2006	2010	Hamdan v. Rumsfeld	Guantanamo Bay	Liberal	30.4%	9.9%	53.2%	38.0%	38.0%	42.3%
2009-2010	2010	Citizens United v. FEC	Campaign Finance	Conservative	44.7%	67.9%	27.4%	64.5%	71.2%	63.7%
2009-2010	2010	U.S. v. Comstock	Sex Offenders	Conservative	54.5%	52.6%	50.1%	33.0%	24.9%	32.6%
2009-2010	2010	Salazar v. Buono	Religious Symbols	Conservative	62.1%	85.4%	43.0%	41.2%	40.4%	39.4%
2007-2008	2010	Crawford v. Marion County	Voter ID	Conservative	81.6%	92.7%	75.3%	71.6%	71.9%	75.5%
2007-2008	2010	Baze v. Rees	Capital Punishment	Conservative	78.8%	93.2%	70.9%	79.5%	76.8%	79.9%
2006-2007	2010	Gonzales v. Carhart	Partial Birth Abortion	Conservative	55.0%	78.6%	37.8%	51.3%	52.2%	50.7%

Table S5. Respondents Views on Individual Cases.

Note: Ideological direction of Court decision defines conservative as the position that is supported at a higher rate by Republican respondents than by Democratic respondents in our survey sample. This choice does not affect our overall ideology estimates since the ideal point model infers ideological direction based on the data.

	% of cases taking liberal position	% of cases Court takes liberal position	% of cases agreeing with Court decision	% of cases correctly predicting Court decision	% of cases respondent prediction matches their own position
**2021 wave**					
Full Sample	52.4%	16.7%	48.0%	50.9%	73.6%
Republicans	39.2%	16.7%	57.6%	52.8%	77.6%
Democrats	64.2%	16.7%	38.8%	50.3%	69.5%
**2020 wave**					
Full Sample	60.4%	70.0%	62.3%	59.4%	78.1%
Republicans	41.3%	70.0%	54.6%	56.5%	83.4%
Democrats	76.5%	70.0%	67.8%	60.6%	72.3%
**2010 wave**					
Full Sample	30.8%	10.0%	65.3%	59.6%	70.2%
Republicans	15.3%	10.0%	76.7%	59.9%	69.6%
Democrats	43.5%	10.0%	57.1%	59.7%	69.3%

Table S6. Summary Statistics of Respondents Views on Cases.

Note: Ideological direction of Court decision defines conservative as the position that is supported at a higher rate by Republican respondents than by Democratic respondents in our survey sample. This choice does not affect our overall ideology estimates since the ideal point model infers ideological direction based on the data.

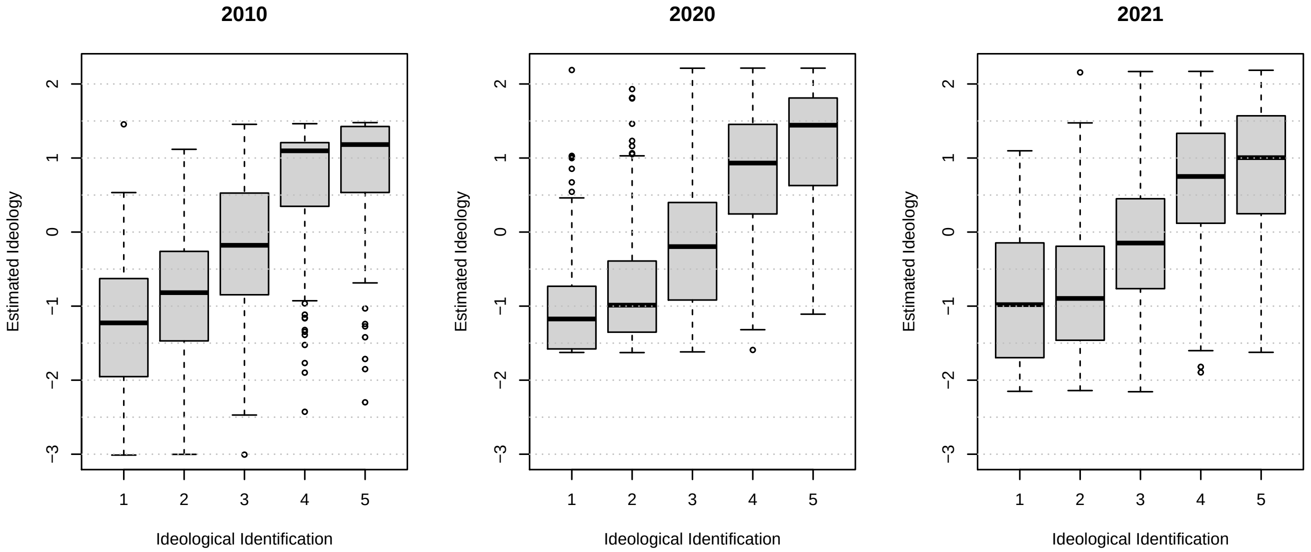

Fig. S1. Boxplot of Estimated Respondent Ideology by Respondent Ideological Identification. *Ideological identification defined by response to question "In general, how would you describe your own political viewpoint?", with responses 1 to 5 corresponding to "Very liberal", "Liberal", "Moderate", "Conservative", and "Very conservative", respectively ("Not sure" responses treated as missing).*

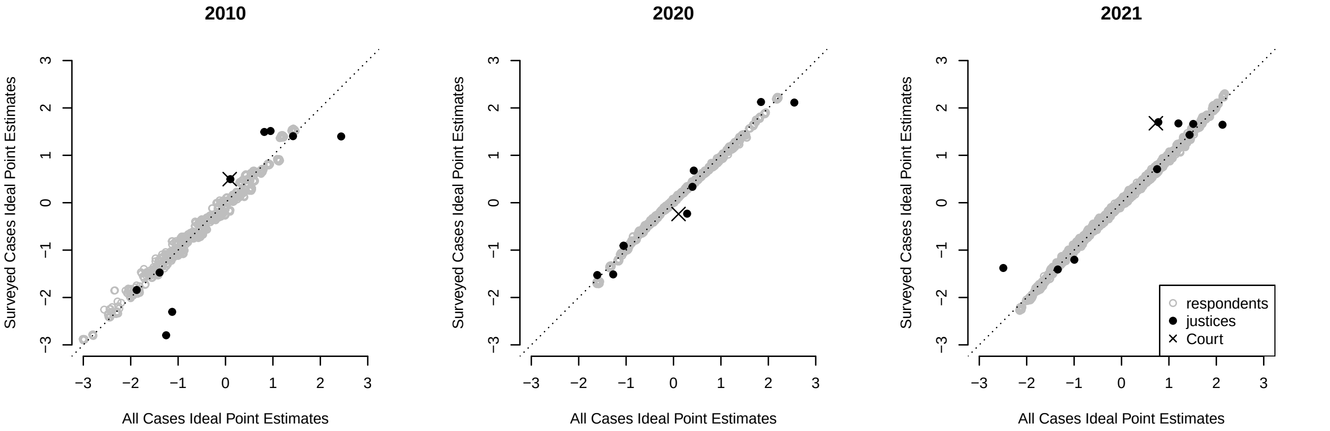

Fig. S2. Comparing Ideology Estimates Using all Cases vs. Using Only Surveyed Cases. *Ideology estimates are similar when using all cases in the Court's docket for each year as compared to using only the cases included in the survey, particularly for respondents. Justice and Court ideology estimates are slightly different due to the "All Cases" estimates being informed by the many cases each term that are not included in the surveys. Information from these cases allows for more precise estimation of justice and Court positions than the "Surveyed Cases" scaling, in which each justice's position (and the Court's position) are quite imprecisely estimated.*

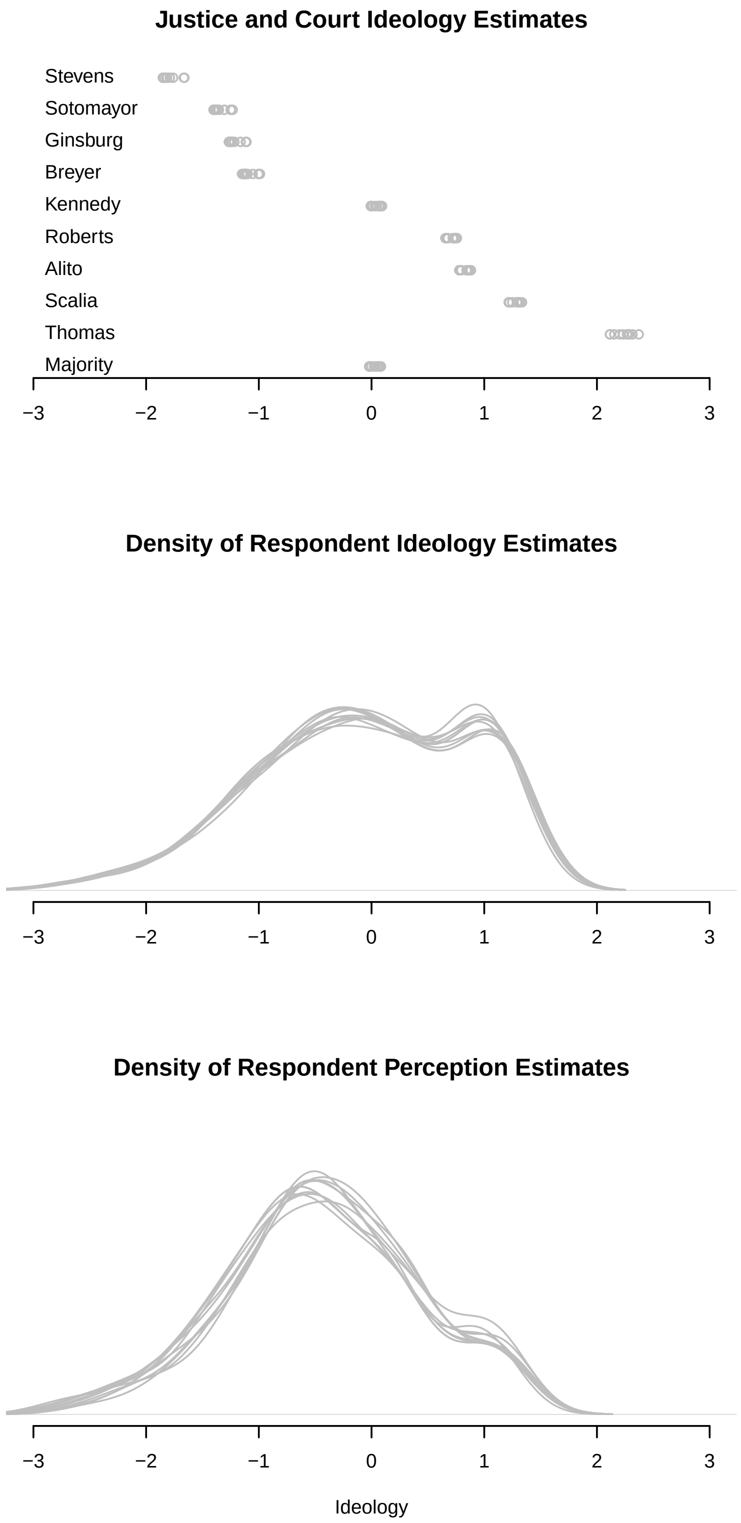

Fig. S3. 2010 Ideology Estimates Dropping One Case at a Time. *Ideology estimates for 2010 wave are similar when leaving out any one of the cases included in the survey from the data. Top pane shows estimates for justices and Court when dropping each individual case (10 sets of estimates are plotted, one for each individual case that is dropped one at a time). Middle (bottom) pane shows density of respondent ideology estimates (estimated respondent perceptions of the Court* from each of these 10 sets of estimates.

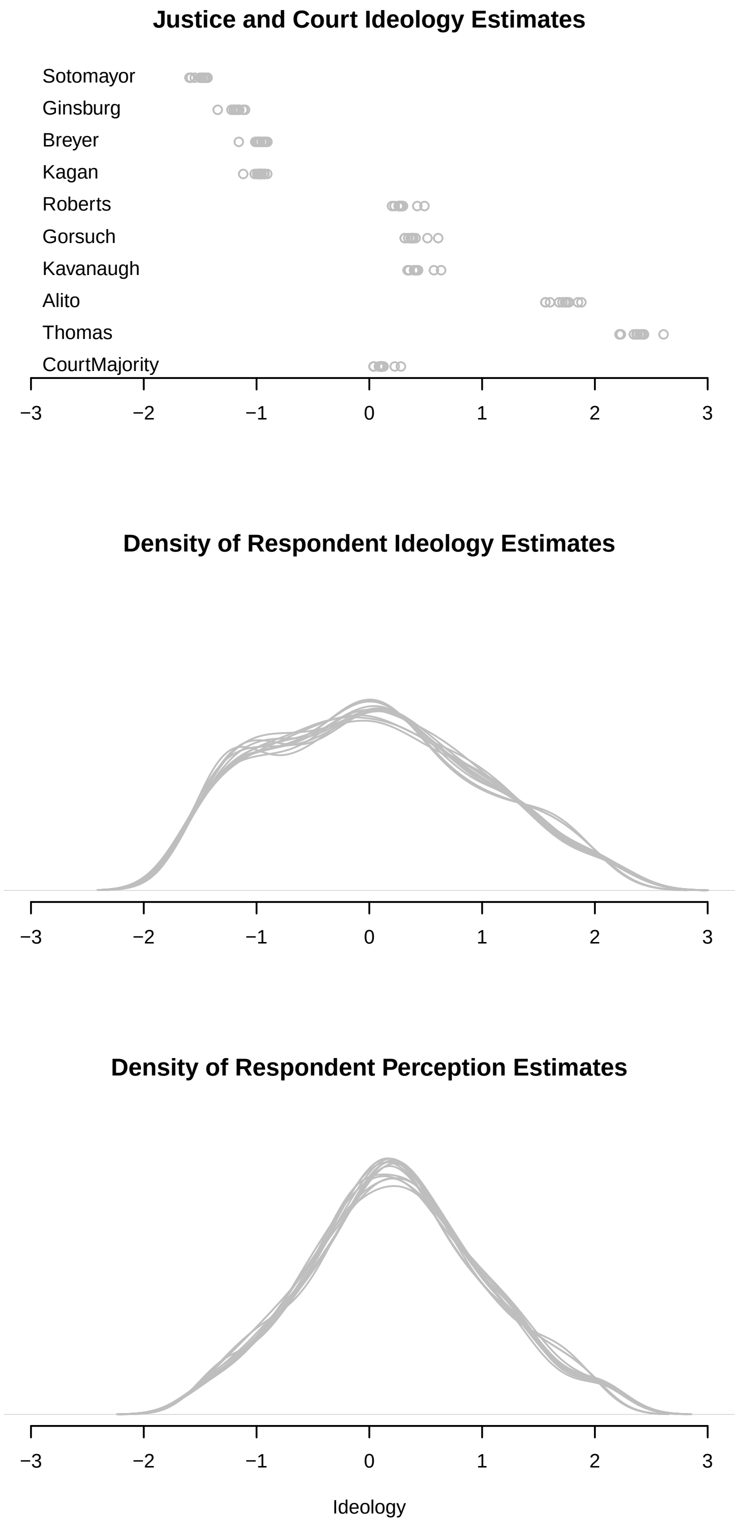

Fig. S4. 2020 Ideology Estimates Dropping One Case at a Time. *Ideology estimates for 2020 wave are similar when leaving out any one of the cases included in the survey from the data. Top pane shows estimates for justices and Court when dropping each individual case (12 sets of estimates are plotted, one for each individual case that is dropped one at a time). Middle (bottom) pane shows density of respondent ideology estimates (estimated respondent perceptions of the Court* from each of these 12 sets of estimates.

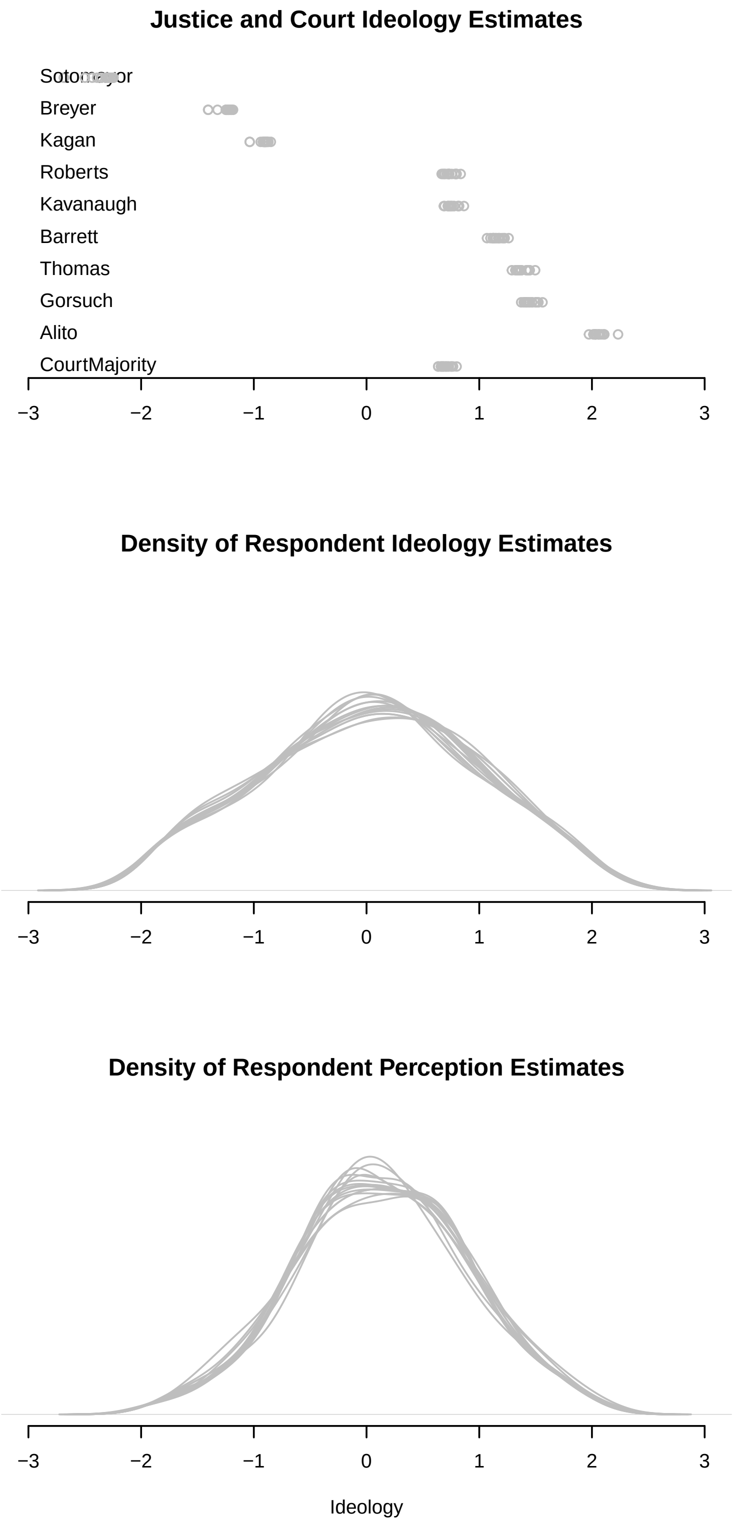

Fig. S5. 2021 Ideology Estimates Dropping One Case at a Time. *Ideology estimates for 2021 wave are similar when leaving out any one of the cases included in the survey from the data. Top pane shows estimates for justices and Court when dropping each individual case (14 sets of estimates are plotted, one for each individual case that is dropped one at a time). Middle (bottom) pane shows density of respondent ideology estimates (estimated respondent perceptions of the Court* from each of these 14 sets of estimates.

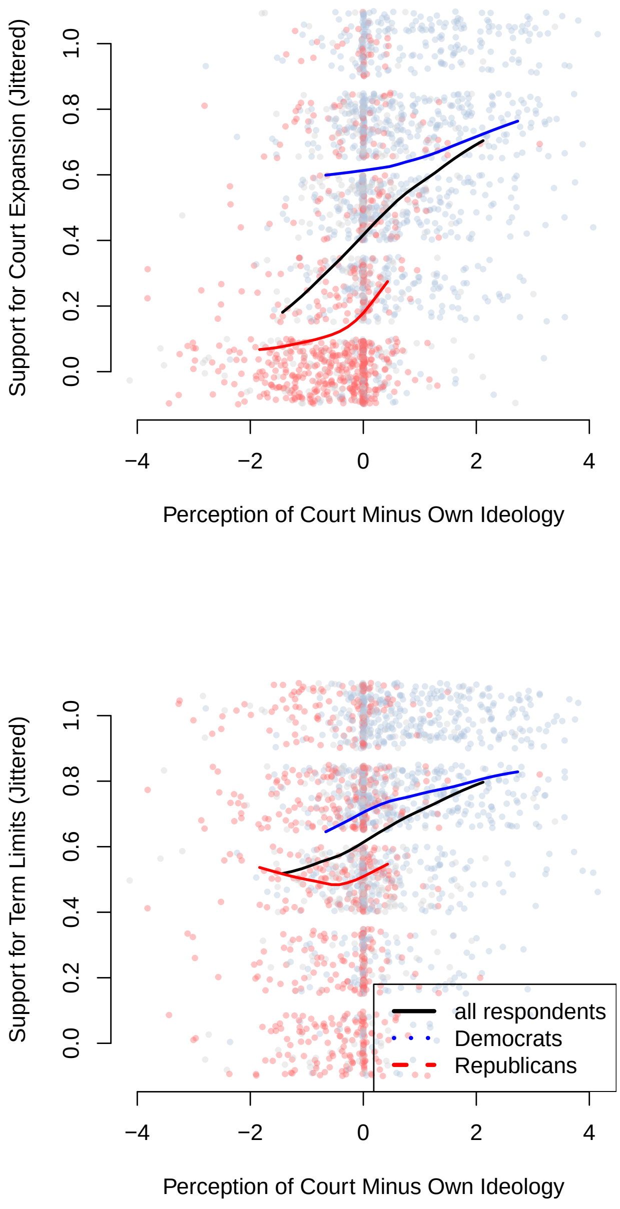

Fig. S6. Predicting Support for Proposed Changes to the Supreme Court with Respondent Ideology. *Red, blue and gray points indicate Republican, Democratic, and independent respondents. Black lines show loess fits using all respondents, while red (blue) lines show linear regression estimates using only Republican (Democratic) respondents.*

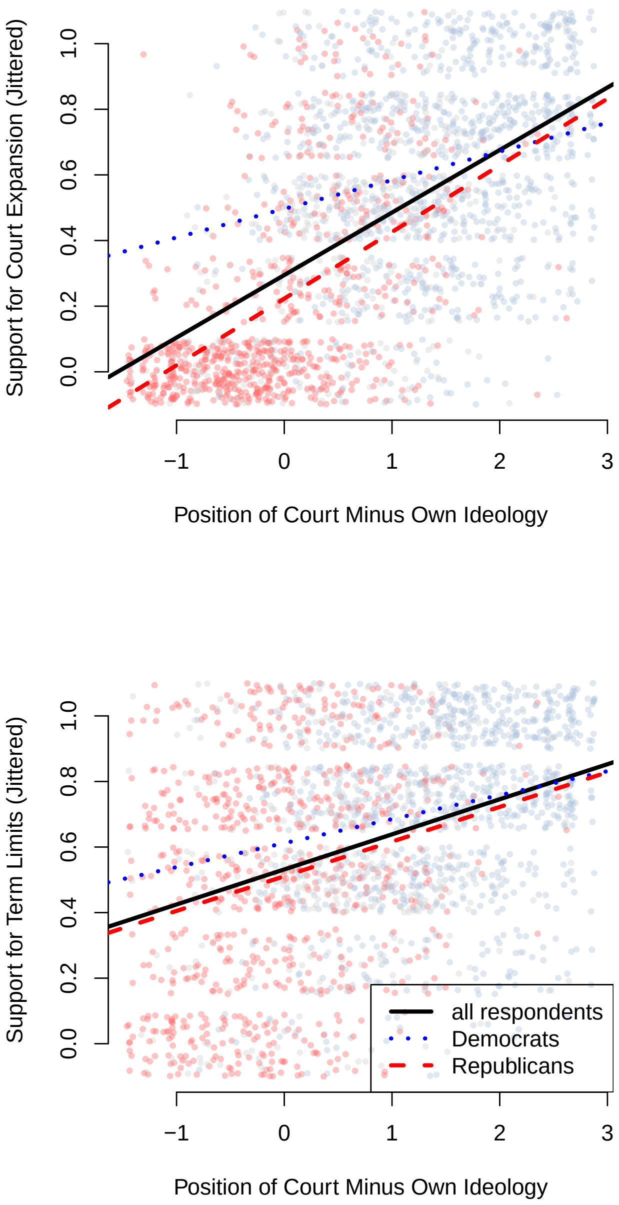

Fig. S7. Predicting Support for Proposed Changes to the Supreme Court with Respondents' Ideological Position Relative to the Court. *Red, blue and gray points indicate Republican, Democratic, and independent respondents. Black lines show linear regression estimates using all respondents, while red (blue) lines show linear regression estimates using only Republican (Democratic) respondents.*

References

1. J Clinton, S Jackman, D Rivers, The statistical analysis of roll call data. *Am. Polit. Sci. Rev.* **98**, 355–70 (2004).
2. S Jackman, et al., Package 'pscl'. *Polit. Sci. Comput. Lab.* **18** (2015).
